# ERK 1/2 Activation Mediates the Neuroprotective Effect of BpV(pic) in Focal Cerebral Ischemia–Reperfusion Injury

**DOI:** 10.1007/s11064-018-2558-z

**Published:** 2018-06-07

**Authors:** Rui Liu, Jun-Chun Tang, Meng-Xian Pan, Yang Zhuang, Ya Zhang, Hua-Bao Liao, Dan Zhao, Yang Lei, Rui-Xue Lei, Shu Wang, An-Chun Liu, Xing-Ping Qin, Juan Chen, Zhi-Feng Zhang, Qi Wan

**Affiliations:** 1 Department of Physiology, Collaborative Innovation Center for Brain Science, School of Basic Medical Sciences, School of Medicine, Wuhan University, 185 Donghu Street, Wuhan, 430071 China; 2 Department of Neurosurgery, Renmin Hospital of Wuhan University, 238 Jiefang Road, Wuhan, Hubei 430060 China; 3 3Department of Neurology, The Central Hospital of Wuhan, Tongji Medical College of Huazhong University of Science & Technology, 26 Shengli Street, Wuhan, 430013 China; 4 Department of Physiology, School of Basic Medical Sciences, Hubei University of Medicine, Shiyan, Hubei 442000 China; 5 Institute of Neuroregeneration & Neurorehabilitation, Department of Neurosurgery of the Affiliated Hospital, Qingdao University, 308 Ningxia Street, Qingdao, 266071 China

**Keywords:** PTEN, BpV(pic), Cerebral ischemia, AKT, ERK 1/2

## Abstract

Bisperoxovanadium (pyridine-2-carboxyl) [bpV(pic)] is a commercially available PTEN inhibitor. Previous studies from us and others have shown that bpV(pic) confers neuroprotection in cerebral ischemia injury. We set up to determine whether ERK 1/2 activation plays a role in bpV(pic)-induced neuroprotective effect in cerebral ischemia injury. We found that the phosphorylation levels of Akt (p-AKT) and ERK1/2 (p-ERK 1/2) were down-regulated after cerebral ischemia–reperfusion injury. The injection of bpV(pic) after injury not only increased the level of p-AKT but also the level of p-ERK 1/2. While the inhibition of PTEN mediated the up-regulatation of p-AKT and p-ERK 1/2 by bpV(pic). Interestingly, the ERK 1/2 activation induced by bpV(pic) was also independent of the inhibition of PTEN. Our results indicate that bpV(pic) protects against OGD-induced neuronal death and promotes the functional recovery of stroke animals through PTEN inhibition and ERK 1/2 activation, respectively. This study suggests that the effect of bpV(pic) on ERK 1/2 signaling should be considered while using bpV(pic) as a PTEN inhibitor.

**Electronic supplementary material:**

The online version of this article (10.1007/s11064-018-2558-z) contains supplementary material, which is available to authorized users.

## Introduction

Ischemic stroke is induced by arterial embolism, microangiopathy or macroaniopathy, results in oxygen and glucose deprivation in brain, leading to brain damage/injury and neurologic deficit [1]. Oxidative stress, inflammation, and excitotoxicity have been considered as major contributors to ischemic neuronal injury [2]. Neuronal injury after cerebral ischemia also produces a complex train of signaling cascades that also lead to cell injury/death [3]. The complex environment and high damage/death ratio after ischemic stroke, neuroprotection becoming a related question. Thrombolytic therapy and neuroprotective drug therapy become two major potential terapeutic strategies after ischemic stroke.

PTEN (phosphatase and tensin homolog deleted on chromosome 10) a tumor suppressor, associate with several neoplastic diseases [4]. PTEN plays an important role in mediating intracellular signaling of cell proliferation and survival [5, 6]. PTEN is a phosphatase has both lipid and protein phosphatase function [7]. But unlike sevaral cellular proteins activated by phosphorylation, PTEN inactivated its phosphorylation through its specific kinases phosphorylate serine and threonine residues in C-terminal region [8]. PTEN depends on its lipid phosphatase activite to inhibit the PI3K/AKT pathway, the PI3K/AKT pathway playing a key role in promoting the cell survival and growth [6, 9]. Substantial evidenced indicate that inhibition of PTEN induces neuroprotection.

Bisperoxovanadium (pyridine-2-carboxyl) [bpV(pic)] is a commercially available PTEN inhibitor [10]. Previous studies from us and others have shown that bpV(pic) confers neuroprotection in cerebral ischemia injury [11]. Bisperoxovanadium (pyridine-2-squaramide) [bpV(pis)], also an inhibitor of PTEN, which was designed by our laboratory. In our study, we found that the bpV(pis) not only can activate AKT activity through PTEN inhibition, but also can enhance ERK 1/2 activation independent of PTEN. The bpV(pis)-induced neuroprotect through both PTEN inhibition and ERK 1/2 activation [12]. we set up to determine whether bpV(pic) exerted its neuroprotective effect in cerebral ischemia injury through both PTEN inhibition and ERK 1/2 activation.

Mitogen-activated protein kinase (MAPK) is a family of serine/threonine protein kinases that are widely expressed in all eukaryotic cells [13]. Extracellular signal-regulated kinase (ERK), one of the extensively studied MAPK family members, is classically associated with receptor protein tyrosine kinase-induced signaling cascades [14]. It consists of two isoenzymes, so namely ERK 1/2, also called p44/42 MAPK. ERK 1/2 is known to mediate neuronal plasticity, migration and cell survival [15, 16]. Up-regulation of ERK 1/2 signalling activity reveals a positive neuroprotective effect in ischaemia stroke models both in vivo and in vitro [17–19].

In this study, we aimed to investigate the relationship of bpV(pic) and ERK 1/2, and confirm the bpV(pic)-induced neuroprotect whether connect with ERK 1/2. Our results show that bpV(pic) confers neuroprotection after cerebral ischemia–reperfusion injury through inhibition of PTEN lipid phosphatase activity as well as ERK 1/2 activation.

## Materials and Methods

### Animals

Adult male Sprague-Dawley (SD) rats were housed with three rats per cage on a 12 h light/dark cycle in a temperature-controlled room (23–25 °C) with free access to water and food. Animals were allowed at least 3 days to acclimatize before experimentation. We used total 248 male rats in our in vivo expriments, 56 male rats were discarded during the operational procedures. 12 adult pregnant female rats and 45 embryos were used in our expriments for cortical neuronal cultures. All animal use and experimental protocols were approved and carried out in compliance with the IACUC guidelines and the Animal Care and Ethics Committee of Wuhan University School of Medicine. Samples to the experimental groups and to collect and process data was assigned by randomization. The experiments were performed by investigators blinded to the groups for which each animal was assigned.

### Focal Cerebral Ischemia and Infarct Measurement

Transient focal cerebral ischaemia was induced using the suture occlusion technique [20, 21]. The whole process do as our lab described [22]. Male Sprague-Dawley rats weighing 250–300 g were anaesthetized with 4% isoflurane in 70% N_2_O and 30% O_2_ by using a mask. The rectal temperature was maintained at 37.0 ± 0.5 °C using a homoeothermic blanket. A midline incision was made in the neck, the right external carotid artery (ECA) was carefully exposed and dissected and a 3–0 monofilament nylon suture was inserted from the ECA into the right internal carotid artery to occlude the origin of the rightmiddle cerebral artery (approximately 22 mm). After 90 min of occlusion, the suture was removed to allow reperfusion, the ECA was ligated and the wound was closed. Sham-operated rats underwent identical surgery and/or i.c.v. injections except that the suture was inserted and withdrawn immediately. At 24 h after middle cerebral artery occlusion (MCAO), rats (n = 24) were reperfused with ice-cold 0.9% saline after anaesthetized with 4% isoflurane in 70% N_2_O and 30% O_2_, and the brains were rapidly removed for western blot analysis and 2,3,5-triphenyltetrazolium chloride (TTC) staining.

The brain was placed in a cooled matrix and 2 mm coronal sections were cut. Individual sections were placed in 10 cm petri dishes and incubated for 30 min in a solution of 2% TTC in phosphate buffered saline at 37 °C. The slices were fixed in 4% paraformaldehyde at 4 °C for 24 h. All image collection, processing and analysis were performed in a blind manner and under controlled environmental lighting. The scanned images were analyzed using image analysis software (Image-Pro Plus Version 6.0, USA). The infarct volume was calculated to correct for edema. The normal volume of contralateral hemisphere and the normal volume of ipsilateral hemisphere were measured, and the infarct percentage was calculated as % contralateral structure to avoid mismeasurement secondary to edema [23, 24].

### Intraventricular Injection (i.c.v) Administration

Rats were anaesthetized with a mixture of 4% isoflurane in 30% O_2_ and 70% N_2_O in a sealed perspective box. When the rats were deeply anaesthetized, we would use the ear bars and upper incisor bar to secure the rat’s head in a stereotaxic frame, and the rats were continuously under anaesthesia with 4% isoflurane using a mask. Next, making a small sagittal incision, and bregma was located as the anatomical reference point. The cerebral ventricle (from the bregma: lateral, 1.5 mm; anteroposterior, − 0.8 mm; depth, 3.5 mm) was performed using a 23 gauge needle attached via polyethylene tubing to a Hamilton microsyringe, and drug infusion at a rate of 1.0 µL/min. Proper needle placement was verified via withdrawing a few microlitres of clear cerebrospinal fluid into the Hamilton microsyringe.

### Cortical Neuron Culture and OGD Insult

The cortical neuronal cultures were prepared from gestation 17 day’s female SD rats [25]. The pregnant rats were killed by cervical dislocation after anaesthetized with 4% isoflurane in 70% N_2_O and 30% O_2_. The embryos were sprayed with 70% ethanol that were removed after the rats. The embryos were rapidly excised, the embryo skull was removed, and the brain tissue was compeletely removed. The cerebral cortex tissue was separated under a microscope and placed in a fresh frozen liquid and placed in ice-cold plating medium (Neurobasal medium, 0.5% FBS, 2% B-27 supplement, 25 mM glutamic acid and 0.5 mM l-glutamax, specifically for culturing primary neurons). The cortical neurons were plated on Petri dishes coated with poly-d-lysine (PDL) and suspended in plating medium. Half of the plating medium was removed and replaced with maintenance medium (Neurobasal medium, 0.5 mM l-glutamine and 2% B-27 supplement) in the same manner every 3 days. The cultured neurons were used for the experiments after 12 days [25].

Oxygen–glucose deprivation (OGD) challenge, cells were transferred to glucose-free extracellular solution (116 mM NaCl, 0.8 mM MgSO_4_, 5.4 mM KCl, 1.0 mM NaH_2_PO_4_, 26 mM NaHCO_3_ and 1.8 mM CaCl_2_) and deoxygenated environment, placed in a humidified chamber (Plas-Labs, Lansing, USA), and maintained at 37 °C in H_2_/5% CO_2_ 85% N_2_/10% for 60 min. Next, replacing with fresh maintenance medium containing an appropriate concentration of reagents for 24 h during the recovery period in a 5% CO_2_/95% O_2_ incubator. The control cultures were firstly transferred to another extracellular solution (5.4 mM KCl, 116 mM NaCl, 0.8 mM MgSO_4_, 1.8 mM CaCl_2_, 1.0 mM NaH_2_PO_4_, 26 mM NaHCO_3_ and 33 mM glucose), and placed in the humidified chamber, which was maintained at 37 °C in 95% O_2_/5% CO_2_ for 60 min [26]. Last, replacing with fresh maintenance medium for the whole period at 37 °C in a 95% O_2_/5% CO_2_ incubator.

### Western Blotting Analysis

Western blotting was performed as previously described [22]. Briefly, the polyvinylidene difluoride (PVDF) membrane by Millipore (USA) was used incubate with a first antibody against ERK 1/2 (Rabbit, 1:2000), phospho-ERK 1/2 (Thr^202^/Tyr^204^) (Rabbit, 1:2000), PTEN (Rabbit, 1:1000), AKT (Mouse, 1:1000), phospho-AKT (Ser^473^) (Rabbit, 1:2000), Actin (Rabbit, 1:2000) from Cell Signaling Technology (MA, USA). First antibodies were labelled with secondary antibody, protein bands were imaged using SuperSignal West Femto Maximum Sensitivity Substrate (Pierce, Rockford, IL, USA). The EC3 Imaging System (UVP, LLC, Uplant, USA) was used to obtained blot images directly from the PVDF membrane. The data of western blot were quantified using Image J software.

### Fluoro-Jade C (FJC) Staining

Rats were treated with an over dose of isoflurane, then intracardiac perfusion with 0.9% saline, next put in 4% paraformaldehyde (PFA) at 4 °C for 24 h, and followed by transfered into 30% sucrose solution in 100 mol/mL phosphate buffer at 4 °C for 72 h. Then the brains tissue were kept in 4% paraformaldehyde solution at 4 °C overnight. Brains tissues were cut into 16 µm coronal sections by a Leica VT1000S vibratome (Leica Micro-systems AG, Nussloch, Germany). FJC labelling was performed using by the standard protocol [27]. Brain sections were first immersed in 1% sodium hydroxide in 80% ethanol for 5 min, then rinsing in 70% ethanol for 2 min, then 2 min in distilled water, and followed by incubated in 0.06% potassium permanganate solution for 10 min. Following a 2 min distilled water rinse, the brain sections were transferred into 0.0001% solution of FJC (Sigma-Aldrich, USA) 10 min which was dissolved in 0.1% acetic acid. Brain sections were rinsed with water for 1 min three times, then air dried on a warmer at 50 °C at least 5 min, last immersed in xylene at least 1 min. The brain sections were mounted with DPX media (Sigma Aldrich, USA). The brain sections were photographed by a blinded investigator using an Olympus fluorescent microscope (IX51, Olympus, Japan). Series of microphotographs were taken from three region of the ipsilateral cerebral cortex, with a ×20 objective and FJC-positive cells were counted by Image J software (ImageJ, USA). The data were expressed as cells/mm^2^.

### Immunofluorescence Analysis

The immunostaining brain sections was prepared as FJC staining, the immunofluorescence staining steps based on the description of the prior execution [28]. The brain sections were treated with primary antibody rabbit anti- phospho-AKT (Ser^473^) (1:250), phospho-ERK 1/2 (Thr^202^/Tyr^204^) (1:250) from Cell Signaling Technology, mouse anti- NeuN (neuronal-specific nuclear protein) from Chemicon. The secondary antibody goat anti- Rabbit 594, goat anti- Mouse 488 from Molecular Probes (Eugene, USA). The hochest probe from Life Technologies. The sections were photographed by a blinded investigator using an Olympus fluorescent microscope (IX51, Olympus, Japan). Analysed by Image J software (Image J, USA).

### Cell Culture, Transfection and Treatment

SH-SY5Y cells and U251 cells were purchased from the Chinese Academy of Sciences Cell Bank. SH-SY5Y cells and U251 cells were sowed in a 6-well plate (8 × 105 cells per well) in DMEM supplemented with 10% heat-inactivated FBS, penicillin G (100 U/mL), streptomycin (100 mg/mL) and l-glutamine (2.0 mM) and incubated at 37 °C in a humidified atmosphere containing 5% CO_2_ and 95% air.

On the day of treatment, when the confluence of SH-SY5Y and U251 cells were reached 80–90%, cells were washed with standard ECS for 60 min and then treated with bpV (pic) for 30 min. The cells were then collected for western blot analysis.

When the confluence of SH-SY5Y cells reached 60–70% on the treatment day, cells were transfected with human PTEN siRNA (siRNApten) and non-targeting control siRNA (NsiRNA) (Santa Cruz Biotechnology, Santa Cruz, CA, USA) for 8 h. The sequence of human PTEN siRNA (siRNApten) was 5′-CTGCTAGCCTCTGGATTTGA-3′ and non-targeting control siRNA (NsiRNA) was 5′-CTTCTGGCATCCGGTTTAGA-3′, as previously described [29]. The medium was then replaced with normal growth medium for 24 h. On the following day, the cells were treated with standard ECS for 60 min and then treated with bpV(pic) for 30 min. The cells were then collected for western blot analysis.

We established the PTEN WT and phosphatase domain mutant: PTEN G129E, in which Glu replaces Gly129, which lacks lipid phosphatase activity (TaiHe Biotechnology Co, LTD) [30].When the confluence of U251 cells reached 60–70% on the treatment day, cells were transfected with human PTEN plasmid pCDNA3.1(+)-PTEN-WT (WT PTEN), pCDNA3.1(+)-PTEN-G129E (PTEN G129E) and pCDNA3.1(+) [Empty vector (EV)] for 8 h. The medium was then replaced with normal growth medium for 24 h. On the following day, the cells were treated with standard ECS for 60 min and then treated with bpV(pic) for 30 min. The cells were then collected for western blot analysis.

### Analysis of LDH Release and Cell Viability

Lactate dehydrogenase (LDH) release was analysed by using a colorimetric CytoTox 96 Cytotoxicity kit (Promega, USA). Cell viability in the neuronal cultures was evaluated by the ability of take up thiazolyl blue tetrazolium bromide (MTT) (PowerWave X, Bio-Tek, Winooski, State, USA). Two methods are following by the manufacturer’s instructions.

### Neurological Severity Scores

The rats were subjected to a modified neurological severity score test as reported previously. These tests are a battery of reflex, sensory, motor and balance tests, which are similar to the contralateral neglect tests in humans. Neurological function was graded on a scale of 0–18 (normal score, 0; maximal deficit score, 18) [31].

### Beam Walk Test

The beam walk test measures the animals’ complex neuromotor function. The animal was timed as it walked a (100 × 2 cm) beam. A box for the animal to feel safe was placed at one end of the beam. A loud noise was created to stimulate the animal to walk towards and into the box. Scoring was based upon the time it took the rat to go into the box. The higher the score, the more severe the neurological deficit [32].

### Adhesive-Removal Test

A modified sticky-tape test was performed to evaluate forelimb function. A sleeve was created using a 3.0 × 1.0 cm piece of yellow paper tape and was subsequently wrapped around the forepaw so that the tape attached to itself and allowed the digits to protrude slightly from the sleeve. The typical response is for the rat to vigorously attempt to remove the sleeve by either pulling at the tape with its mouth or brushing the tape with its contralateral paw. The rat was placed in its cage and observed for 30 s. Two timers were started: the first ran without interruption and the second was turned on only while the animal attempted to remove the tape sleeve. The ratio of the left (affected)/right (unaffected) forelimb performance was recorded. The contralateral and ipsilateral limbs were tested separately. The test was repeated three times per test day, and the best two scores of the day were averaged. The lower the ratio, the more severe the neurological deficit [33].

### Statistics

In this study, on experimental design and analysis, the data and statistical analysis comply with the recommendations. All datas are expressed as mean ± SE. Student’s t-test and variance analysis was used in differences among groups. *P* < 0.05 was considered statistically significant.

## Materials

Bisperoxovanadium (pyridine-2-carboxyl) [bpV(pic)] and AKT Inhibitor IV (ChemBridge 5233705) were purchased from Santa Cruz Biotechnology (Santa Cruz, CA). ERK 1/2 inhibitor U0126 was purchased from Delleckchem (Houston, TX).

## Results

### The Level of Phospho-AKT and Phospho-ERK 1/2 are Down-Regulated After Cerebral Ischemia–Reperfusion Injury

**Fig. 1:**
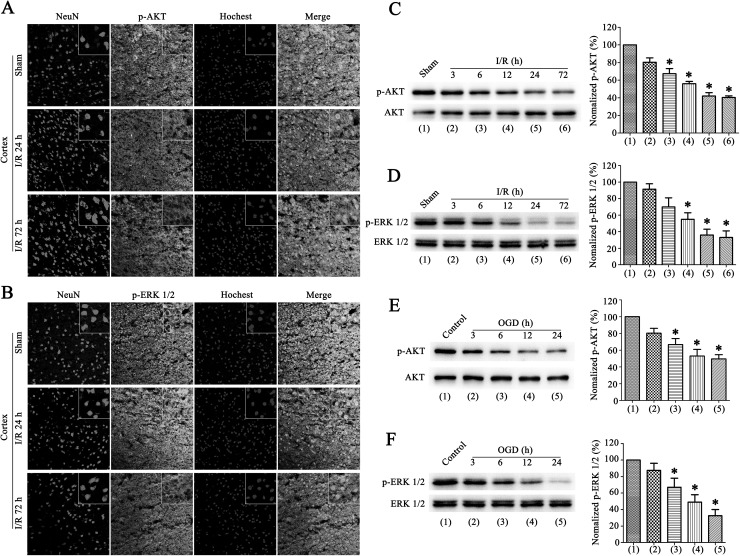
After ischemic stroke p-AKT and P-ERK 1/2 levels are decreased. **a** and **b** Double-immunofluorescence staining of p-AKT or p-ERK 1/2 with NeuN in the peri-infarct area of cortex 24 h or 72 h after I/R compared with the ipsilateral sham, NeuN performes green, P-AKT and p-ERK 1/2 is shown in red and hochest is shown in blue. Scale bar, 20 µm. **c** and **d** Western blots showing a decreasing expression in p-AKT (**c**) and p-ERK 1/2 (**d**) at the indicated time points after I/R at rats (left). Right: quantification analysis of normalized p-AKT and p-ERK 1/2 levels (n = 6 per time points, *P < 0.05 vs. the sham). **e** and **f** Western blots of p-AKT (**e**) and p-ERK 1/2 (**f**) in OGD-treated cultured primary neurons (left) and quantification analysis of p-AKT and p-ERK 1/2 levels (right), showing a similar down-regulation with rats sample (n = 6, *P < 0.05 vs. the control). The data are expressed as mean ± SE. Statistical analysis was implemented by student’s t-test and variance analysis. (Color figure online)

Ischemic stroke results from thrombotic blockage, which in brain causes the oxygen and glucose deprivation, leading to brain damage and neurological deficit. We generated an experimental stroke model induced by MCAO [34, 35] for 1.5 h followed by various periods of reperfusion to simulate ischemia stroke. AKT activation was quantified by measuring AKT phosphorylation (p-AKT) on Ser^473^ [25] and the activation of ERK 1/2 was measured by quantifying ERK 1/2 phosphorylation (p-ERK 1/2) on Thr^202^/Tyr^204^ [36]. Immunofluorescence staining of p-AKT and p-ERK 1/2 in the peri-infarct area of cortex after ischemia–reperfusion (I/R), results show that a decrease p-AKT and p-ERK 1/2 signals were obtained after I/R compared with sham (Fig. 1a, b). Neurons are more vulnerable to stroke insult than other cells in the brain [3]. Double-immunofluorescence labelling detected a down-regulate of p-AKT and p-ERK 1/2 signal in neurons, and did not show any significant difference at I/R 24 h or 72 h (Fig. 1a, b).

Western boltting analysis of ipsilateral brain homogenates at the indicated time point after I/R revealed a down-regulate p-AKT and p-ERK 1/2 levels as time goes on (Fig. 1c, d). Furthermore, we cultured primary neurons from the rat cortex, and used a OGD insult model. Western boltting analysis of p-AKT and p-ERK 1/2 in cultured cortical neurons after OGD insult, results showed a similar p-AKT and p-ERK 1/2 levels pattern with the animals (Fig. 1g, h). These data shows that the neuronal p-AKT and p-ERK 1/2 are down-regulated after ischemic stroke.

### BpV(pic) Induces Not Only the Level of P-AKT Up-Regulation But Also P-ERK 1/2 and Protects Against Ischemia-Induced Brain Damage and Neuronal Death

**Fig. 2:**
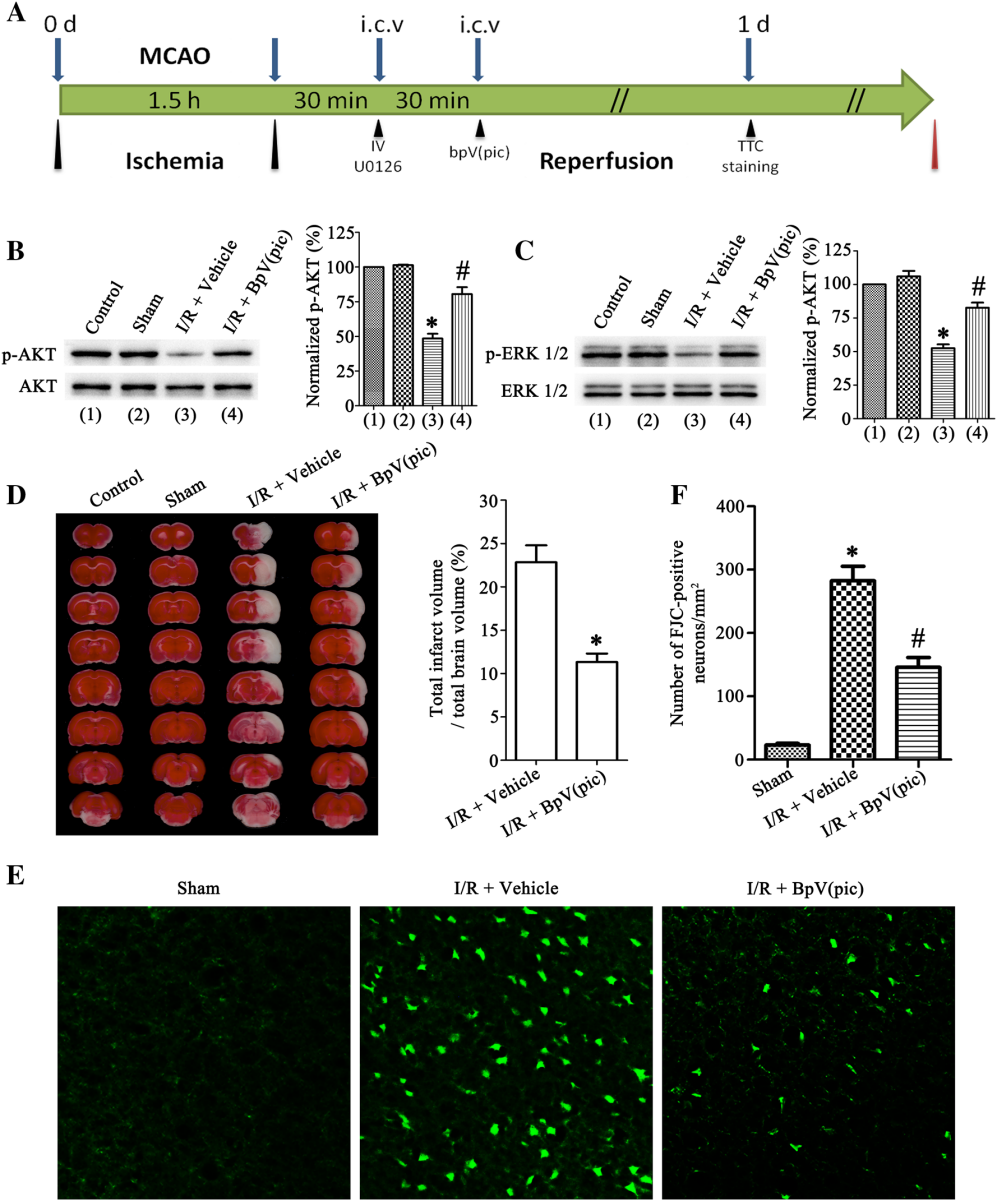
BpV(pic) up-regulated the p-AKT and p-ERK 1/2 level in rats and protects against ischemia–reperfusion injury. **a** A time points diagram shows rat ischemia–reperfusion injury and IV (AKT inhibitor), U0126 (ERK 1/2 inhibitor), bpV(pic) treatment procedure. **b** and **c** Western blots showing an increased expression in p-AKT (**b**) and p-ERK 1/2 (**c**) after i.c.v inject bpV(pic) (100 µM, 5 µL) 24 h after ischemia–reperfusion injury comparing with I/R + vehicle group (left). Right: quantification analysis of p-AKT and p-ERK 1/2 levels (n = 6, *P < 0.05 vs. the sham, ^#^P < 0.05 vs. the I/R + vehicle). **d** Sample images of TTC staining brain sections show that bpV(pic) decreases the infarct volume in brain 24 h after ischemia onset comparing with I/R + vehicle group (left), and quantification analysis of the infarct volume on right (n = 6, *P < 0.05 vs. the I/R + vehicle). **e** Fluoro-Jade C (FJC) staining brain sections collected at 24 h after ischemia onset. BpV(pic) was injected at 1 h after MCAO. Scale bar, 100 µm. **f** Quantitative analysis of Fluoro-Jade C positive degeneration neurons shows an up-regulation numbers after I/R compare with sham, and presents a positive down-regulation while is injected bpV(pic) (n = 6, *P < 0.05 vs. the sham, ^#^P < 0.05 vs. the I/R + vehicle). The datas are expressed as mean ± SE. Statistical analysis was implemented by student’s t-test and variance analysis

BpV(pic) can activate AKT through inhibition of PTEN. Previous studies have shown that bpV(pic) confers neuroprotection in cerebral ischemia injury [11]. In the Fig. 1, we have shown that the level of p-AKT decreased after I/R, therefore, we used the PTEN inhibitor bpV(pic) to treat the animals. Rats were given bpV(pic) at a dose of 100 µM (5 µL) 1.0 h after MCAO by intracerebroventricular (i.c.v) injection (Fig. 2a). Rats in control group received i.c.v injection of vehicle (0.9% saline). Western blotting analysis of p-AKT after I/R 24 h showed that the level of p-AKT up-regulated after bpV(pic) treatment (Fig. 2b). Interestingly, the level of p-ERK 1/2 also up-regulated (Fig. 2c). To further confilm the neuroprotective of bpV(pic), TTC straining analysis was performed in the mouse brain tissues, results show that the bpV(pic) was against the ischemia-induced brain damage (Fig. 2d). Further, we used the Fluoro-Jade C (FJC) straining to determine the neurons status. Results show that the number of FJC-positive degenerating neurons in the cortex of peri-infarct area were significantly decreased after bpV(pic) treatment (Fig. 2e, f). These results further comfirm the neuroprotective effect of bpV(pic) in the neurons. Together, we confilm the neuroprotective effect of bpV(pic) in cerebral ischemia injury, and we further reveal that the bpV(pic) induces not only the level of p-AKT up-regulation but also p-ERK 1/2.

### BpV(pic)-Mediated AKT Activation Get Through by Inhibition of PTEN Lipid Phosphatase Activity

**Fig. 3:**
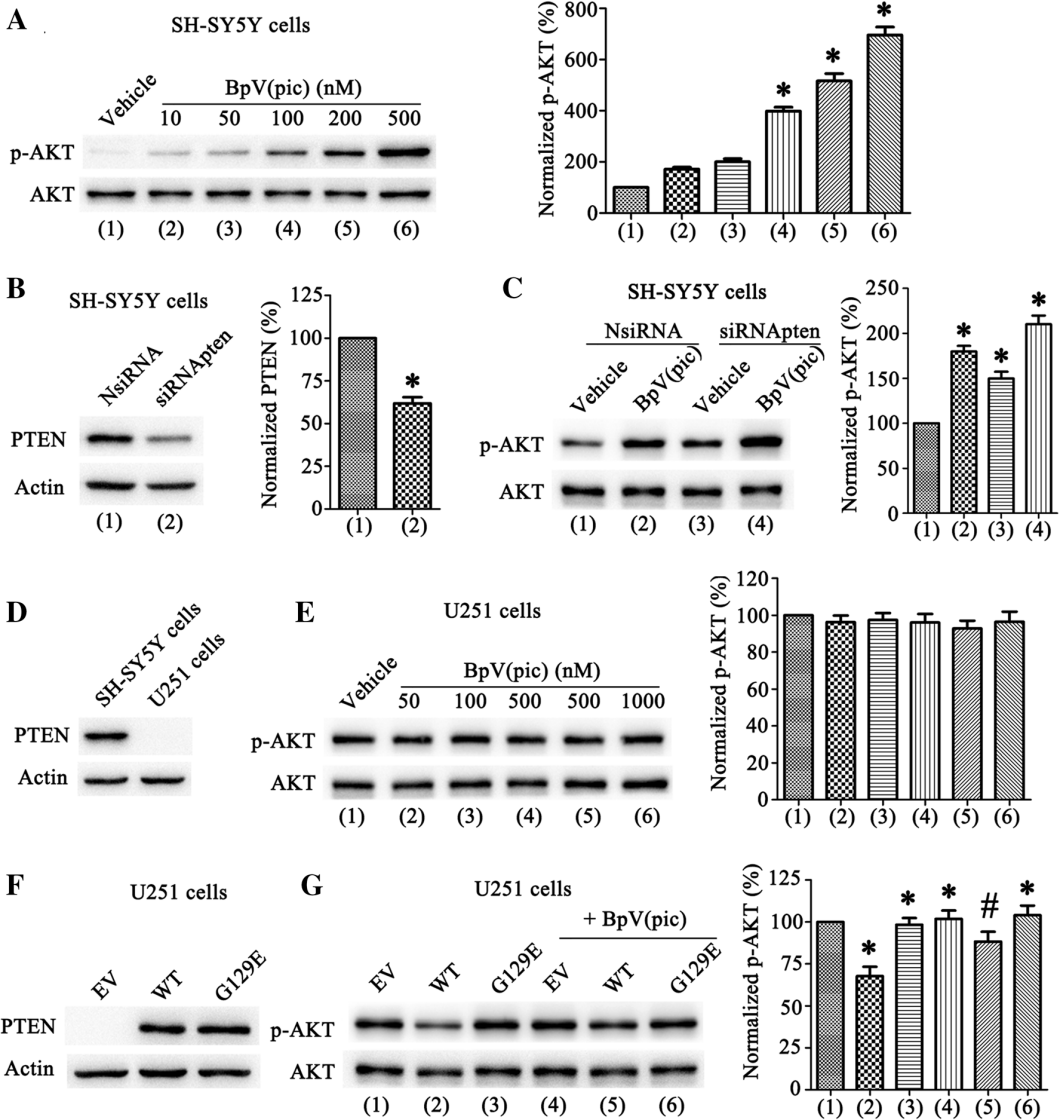
BpV(pic) up regulates p-AKT level through inhibiting PTEN lipid phosphatase activity. **a** Western blots analysis of p-AKT levels in SH-SY5Y cells treated with bpV(pic) (10–500 nM) in the right. Left: quantification analysis of p-AKT levels treated with bpV(pic) shown an increased expression of normalized p-AKT compare with vehicle group (n = 6 independent cultures, *P < 0.05 vs. the vehicle). **b** In SH-SY5Y cells PTEN expression decreased after transfected with siRNApten (n = 6 independent cultures, *P < 0.05 vs. the NsiRNA). **c** The p-AKT levels in SH-SY5Y cells transfect with NsiRNA or siRNApten then treated with bpV(pic) (200 nM) (n = 6 independent cultures, *P < 0.05 vs. the NsiRNA + vehicle). **d** Western blots analysis of PTEN expression in SH-SY5Y and U251 cells. **e** The level of p-AKT in U251 cells treatment with bpV(pic) (50–1000 nM) did not change (n = 6 independent cultures). **f** Western blots of PTEN expression in U251 cells transfected with PTEN-cDNA WT and G129E. **g** The levels of p-AKT in U251 cells transfected with PTEN-cDNA WT and G129E, then treatment with bpV(pic) (200 nM) (n = 6 independent cultures, *P < 0.05 vs. the EV, ^#^P < 0.05 vs. the WT). The data are expressed as mean ± SE. The datas are expressed as mean ± SE. Statistical analysis was implemented by student’s t-test and variance analysis

To further investigate the effect of bpV(pic). We used the human neuroblastoma SH-SY5Y cells, which were treated with different concentrations of bpV(pic) (10, 50, 100, 200 and 500 nM). Results show that bpV(pic) increased the level of p-AKT in SH-SY5Y cells (Fig. 3a). BpV(pic) mostly performs as a PTEN inhibitor to activate AKT activity, therefore, we used a PTEN knockdown approach in SH-SY5Y cells (Fig. 3b) to further confilm the relationship of PTEN with AKT activation. Results show that the level of p-AKT increased after PTEN consumption (Fig. 3c). Moreover, the bpV(pic) (200 nM) was traeated in SH-SY5Y cells after transfection of siRNApten, bpV(pic) did not cause a significant increase of p-AKT level in SH-SY5Y cells after transfection of siRNApten (Fig. 3c). To further investigate whether the bpV(pic)-induced p-AKT up-regulation depends only on PTEN. We used a PTEN-deficient cell line, human glioblasoma U251 cells (Fig. 3d). The level of p-AKT did not up-regulation after bpV(pic) treatment (Fig. 3e). Together, these results indicate that the bpV(pic) enhances AKT activation by inhibition of PTEN.

We know from a previous study that PTEN can negatively regulate AKT activation through PI3K signal pathway because of it’s lipid phosphatase activity. To further reveal the mechanism of bpV(pic) inhibition function in PTEN, we established the PTEN WT and phosphatase domain mutant: PTEN G129E, in which Glu replaces Gly129, which lacks lipid phosphatase activity [30]. Because U251 cell line has no PTEN expression, we first confirmed that PTEN-cDNA was expressed in U251 cells (Fig. 3f). Next, we tested the level of p-AKT after transfected with EV, WT or G129E. The resultsshow that the level of p-AKT was down-regulated in WT group compared with EV, and did not show any significant changes after transfection of PTEN G129E (Fig. 3g). Furthermore, we treated bpV(pic) (200 nM) in U251 cells after transfection of PTEN plasmid. Results show that the level of p-AKT did not increase in the EV + bpV(pic) and G129E + bpV(pic) group compared with EV, however, increased in the WT + bpV(pic) group compared with WT group (Fig. 3g). These results show that bpV(pic) increases AKT activation through inhibiting PTEN lipid phosphatase activity.

### BpV(pic) Increases ERK 1/2 Activition Through Both Inhibition of PTEN Lipid Phosphatase and Independent of PTEN

**Fig. 4:**
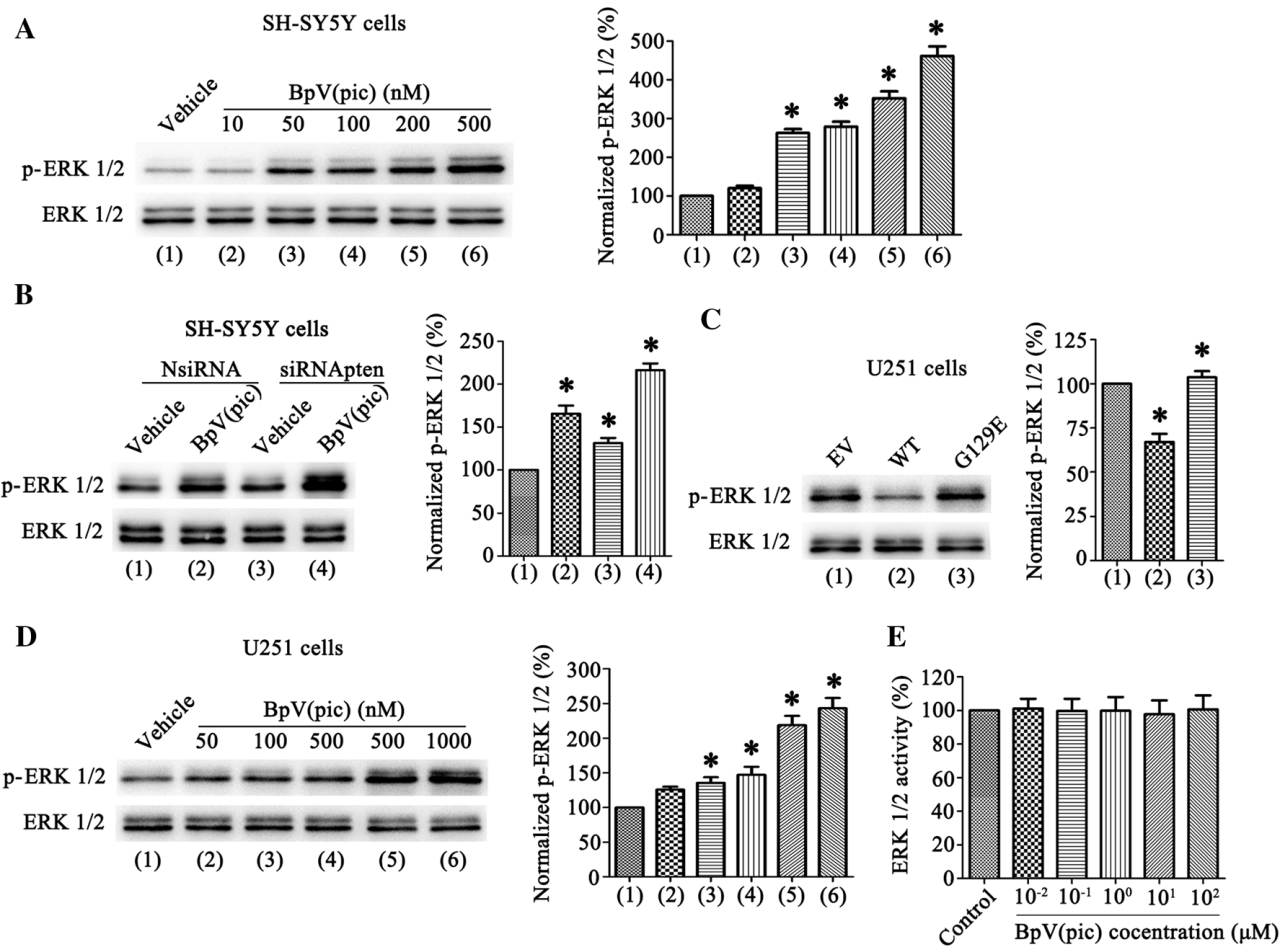
BpV(pic) not only through inhibit PTEN lipid phosphatase activity but also independently of PTEN to up-regulation p-ERK 1/2 level. **a** Western blots analysis of p-ERK 1/2 levels in SH-SY5Y cells treated with bpV(pic) (10–500 nM) on right. Left: quantification analysis of p-ERK 1/2 levels treated with bpV(pic) shows an increased expression of normalized p-ERK 1/2 compare with vehicle group (n = 6 independent cultures, *P < 0.05 vs. the vehicle). **b** The p-ERK 1/2 levels in SH-SY5Y cells transfected with NsiRNA or siRNApten then treated with bpV(pic) (200 nM) (n = 6 independent cultures, *P < 0.05 vs. the NsiRNA + vehicle). **c** The levels of p-ERK 1/2 in U251 cells transfected with PTEN-cDNA WT and G129E (n = 6 independent cultures, *P < 0.05 vs. the EV). **d** The levels of p-ERK 1/2 increased in PTEN-deficient cell U251 cells when treated with bpV(pic) (50–1000 nM). Quantification analysis of p-ERK 1/2 levels on the right (n = 6 independent cultures, *P < 0.05 vs. the vehicle). **e** the ERK 1/2 levels have no change by bpV(pic) in vitro (n = 6 independent cultures). The data is expressed as mean ± SE. Statistical analysis was implemented by student’s t-test and variance analysis

From Fig. 2c, we found that the level of p-ERK 1/2 increased after bpV(pic) treatment in rats. To further inverstigate the mechanism of bpV(pic) regulates ERK 1/2 activation, we firstly increased the level of p-ERK 1/2 in SH-SY5Y cells after treatment different concentrations of bpV(pic) (Fig. 4a). Furthermore, we also measured the effect of PTEN knockdown on ERK 1/2 phosphorylation in SH-SY5Y cells. After PTEN suppression, we found the level of p-ERK 1/2 was increased (Fig. 4b). These results show that the ERK 1/2 activation can be mediate by PTEN. Moreover, we tested the level of p-ERK 1/2 in U251 cells after transfection of PTEN plasmid, results show that the level of p-ERK 1/2 decreased in WT group and did not show any significant difference in G129E group compared with EV (Fig. 4c). These results indicate that the ERK 1/2 can be activared by inhibition PTEN lipid phosphase activity. We further tested the effect of bpV(pic) (50, 100, 200, 500 and 1000 nM) in U251 cells, interestingly, the level of p-ERK 1/2 also up-regulated (Fig. 4d). These data indicate that bpV(pic) activates ERK 1/2 can independently of PTEN. Together, BpV(pic) increases ERK1/2 activition through both inhibition of PTEN lipid phosphatase and independent of PTEN.

We set up to determine whether bpV(pic) directly activates of ERK 1/2, so we used the recombinant ERK 1/2 to test the activation effect of bpV(pic) in it [12]. Our results did not show alterations in the ERK 1/2 activity (Fig. 4e), from which we know that bpV(pic) can not directly increase ERK 1/2 activity.

### BpV(pic) Protects Against OGD-Induced Neuronal Death Through Both AKT and ERK 1/2 Activation

**Fig. 5:**
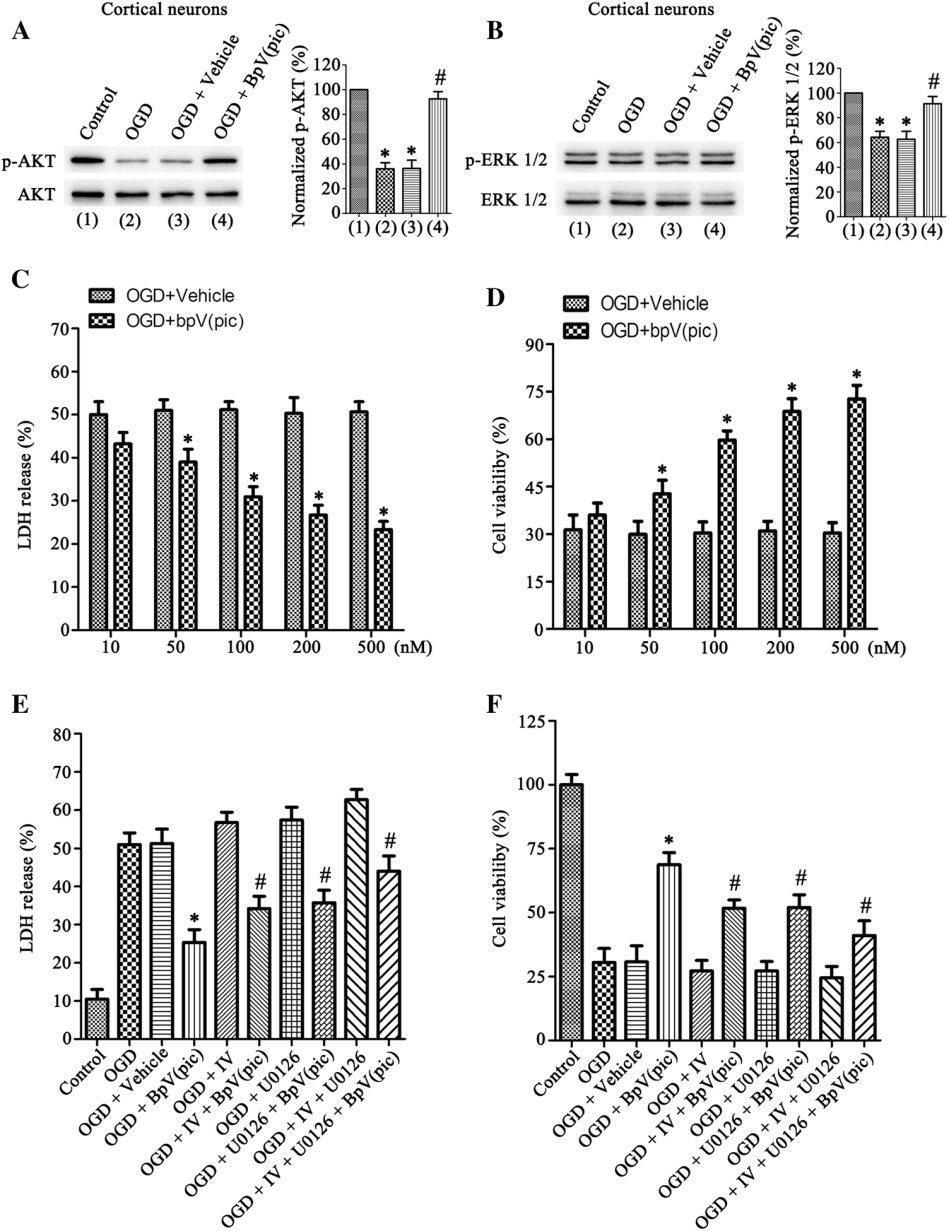
BpV(pic) protect against OGD induced neuronal death through ERK 1/2 activation and PTEN lipid phosphatase activity inhibition. **a** and **b** Western blots analysis of p-AKT (**a**) and p-ERK 1/2 (**b**) levels in cultured primary neurons, bpV(pic) (200 nM) againsts the OGD-induced p-AKT and p-ERK 1/2 down-regulation. Quantification analysis of the levels are on the right (n = 6 independent cultures, *P < 0.05 vs. the control, ^#^P < 0.05 vs. the OGD + vehicle). **c** and **d** During OGD induced neuronal death the LDH release (**c**) decreased and the cell viability (**d**) increased after treated with bpV(pic) (10–500 nM). This tests were performed in quintuplicate (n = 6 independent cultures, *P < 0.05 vs. the OGD + Vehicle). **e** and **f** IV and/or U0126 were treated 30 min after re-oxygenation, bpV(pic) was treated for 1.0 h followed by re-oxygenation in the cultured primary neurons. The LDH release (**e**) and the cell viability (**f**) results show that the neuroprotective effect of bpV(pic) was blocked by IV and U0126 [n = 6 independent cultures, *P < 0.05 vs. the OGD + Vehicle, ^#^P < 0.05 vs. the OGD + bpV(pic)]. The data are expressed as mean ± SE. Statistical analysis was implemented by student’s t-test and variance analysis

From Fig. 1e, f, we found the level of p-AKT and p-ERK 1/2 decreased in the cultured cortical neurons after OGD insult. We up-regulated the level of p-AKT and p-ERK 1/2 in cultured cortical neurons after 24 h of OGD insult (Fig. 5a, b). Furthermore, we used LDH and MTT assay measure neuronal death/viability in cultured cortical neurons after 24 h of OGD insult. Results show that the bpV(pic) decreased LDH release and increased cell viability of neurons (Fig. 5c, d). We have confirmed that bpV(pic) can activation both of AKT and ERK 1/2, an AKT inhibitor IV (1.0 µM) and/ or an ERK 1/2 inhibitor U0126 (10 µM) were treated in the cultured cortical neurons 30 min after re-oxygenation, then the bpV(pic) (200 nM) was treated 1 h after re-oxygenation, we obtained a down-regulation neuroprotective effect of bpV(pic) after inhibition of AKT and/or ERK 1/2 (Fig. 5e, f). Therefore, BpV(pic) protects against OGD-induced neuronal death through both AKT and ERK 1/2 activation.

### BpV(pic) Reduces the Infarct Volume and Promotes Functional Recovery in Ischemic Stroke Animals Through AKT and ERK 1/2 Activation

**Fig. 6:**
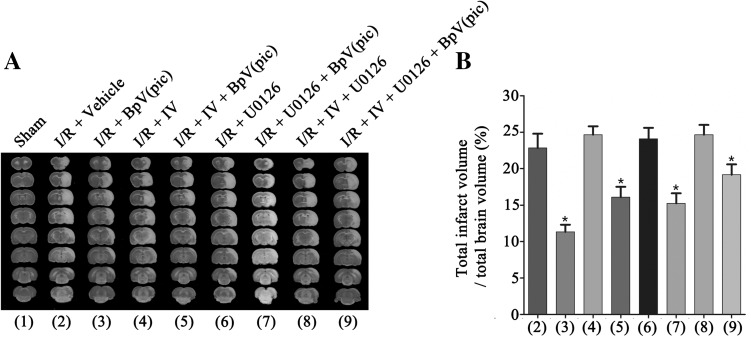
BpV(pic) through PTEN inhibition and ERK 1/2 activation reduces the infarct volume in ischemic stroke animals. **a** Sample images of TTC staining brain sections show that bpV(pic) decreases the infarct volume in brain 24 h after ischemia onset was prevented by IV and U0126. **b** Quantification analysis of the infarct volume [n = 6, *P < 0.05 vs. the I/R + bpV(pic)]. The data are expressed as mean ± SE. Statistical analysis was implemented by student’s t-test and variance analysis

**Fig. 7:**
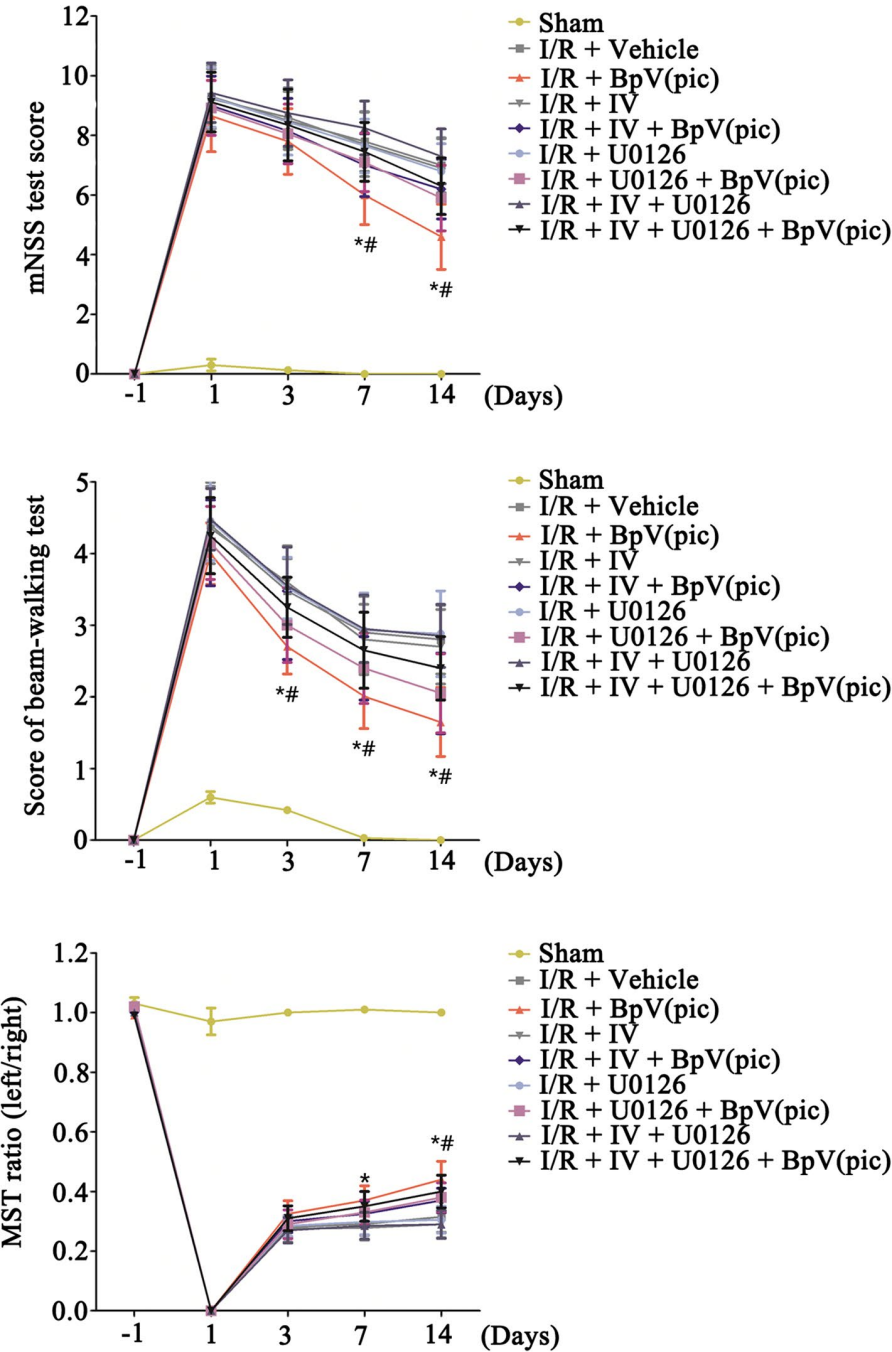
BpV(pic) induces the functional recovery in ischemic stroke animals through PTEN inhibition and ERK 1/2 activation. **a** Animals treated with bpV(pic) have lower scores in mNSS test at day 7 and 14 after ischemia–reperfusion injury compared with I/R + Vehicle group. Animals injected with IV and/or U0126 before injected with bpV(pic) show a higher scores in mNSS test at day 7 and 14 after ischemia–reperfusion than I/R + bpV(pic) group [n = 6 for each group, *P < 0.05 vs. the I/R + Vehicle,^#^P < 0.05 vs. the I/R + bpV(pic)]. **b** Animals treated with bpV(pic) have lower scores in beam-walking test at day 3, 7 and 14 after ischemia–reperfusion injury compared with I/R + Vehicle group. Animals injected with IV and/or U0126 before injected bpV(pic) show a higher scores in beam-walking test at day 3, 7 and 14 after ischemia–reperfusion than I/R + bpV(pic) group [n = 6 for each group, *P < 0.05 vs. the I/R + Vehicle,^#^P < 0.05 vs. the I/R + bpV(pic)]. **c** Animals treated with bpV(pic) have a highter ratio in MST test at day 14 after ischemia–reperfusion injury compared with I/R + Vehicle group. Animals injected with IV and/or U0126 before injected bpV(pic) show lower ratio in MST test at day 14 after ischemia–reperfusion than I/R + bpV(pic) group [n = 6 for each group, *P < 0.05 vs. the I/R + Vehicle,^#^P < 0.05 vs. the I/R + bpV(pic)]. The datas are expressed as mean ± SE. Statistical analysis was implemented by student’s t-test and variance analysis

On the basis of Fig. 5e, f, we further confirmed the neuronprotective effect role of bpV(pic) in vivo. Followed the Fig. 2a, we i.c.v injected IV (100 µM, 2 µL) and/or U0126 (500 µM, 2 µL) 30 min after MCAO, and bpV(pic) (100 µM, 5 µL) was i.c.v injected 1.0 h after MCAO [37, 38]. Results show that inhibition of AKT and/or ERK 1/2 activation prevented the bpV(pic)-induced infarct volume down-regulation (Fig. 6a, b). To further provide evidence for the bpV(pic)-induced functional recovery after MCAO model rats, we performed a train of neurobehavioral tests. As show in Fig. 7a–c, results show that bpV(pic)-induced stroke animals functional recovery were mediated through both AKT and ERK 1/2 signalling pathways.

## Discussion

PTEN is widely known as a tumor suppressor that is mostly associated with several neoplastic diseases. PTEN also plays a pivotal role in neuropathic diseases [39, 40]. Previous study shows that PTEN have both protein and lipid phosphatase activity [7]. When the PTEN protein phosphatase activity is suppressed, the extrasynapic GluN2B containing NMDA receptors (NMDARs) would be inhibited [41]. While inhibiting the lipid phosphatase activity of PTEN, the AKT pathway is activated. No matter which of the phosphatase activity is inhibited, it would induce neuroprotection in ischemic stroke. We further showed that inhibiting PTEN induces the nuclear TDP-43 (TAR DNA-binding protein-43) increase [42], and also produce the GABAA receptor expression and function enhancement to protect against the ischemic stroke induced neuronal death in vivo and in vitro [43, 44]. PTEN inhibition-induced neuroprotection is also confirmed by other studies [45–47].

**Fig. 8:**
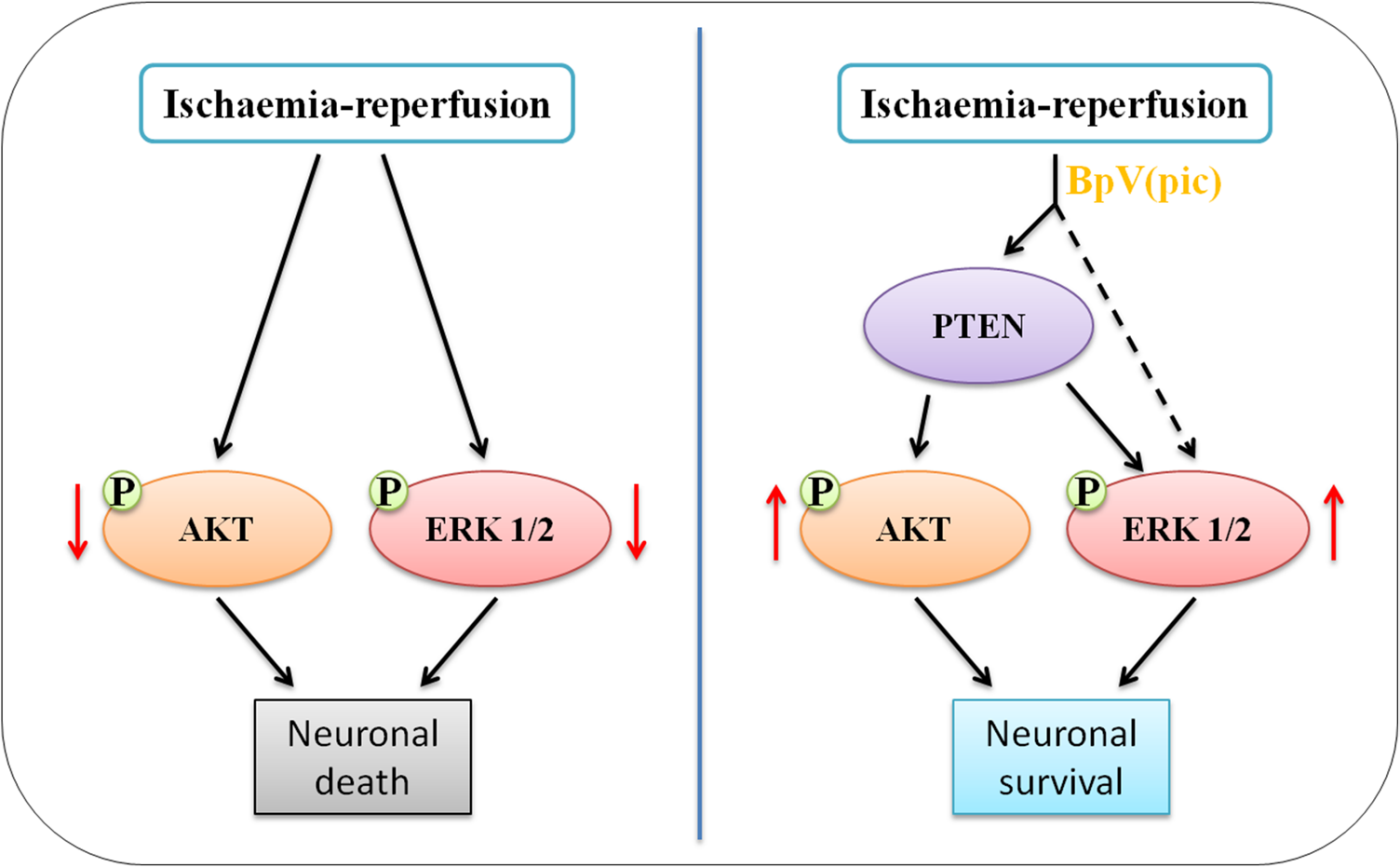
The mechanism of bpV(pic)-mediateded neuroprotect in ischaemia–reperfusion cerebral injury. After ischaemia–reperfusion, the phospho-AKT (Ser^473^) and phospho-ERK 1/2 (Thr^202^/Tyr^204^) were down-regulated, inducing the increase of neuronal death and cerebral injury (left). When treated with bpV(pic), we found that bpV(pic) can not only enhance the level of p-AKT and p-ERK 1/2 through inhibiting PTEN lipid phosphatase activity, but also in a PTEN independent pathway to up regulation of ERK 1/2 activity, leading to neuronal survival and animal functional recovery

BpV(pic) is a commercially available PTEN inhibitor, and previous studies from us and others have shown that bpV(pic) confers neuroprotection in cerebral ischemia injury [47, 48]. We have recently reported a new compound, the bisperoxovandium (pyridin-2-squaramide) [bpV(pis)], an inhibitor of PTEN, the structure is similar to bpV(pic), that confers neuroprotection in the ischemic injury model in vitro and in vivo through suppressing PTEN and activating ERK 1/2 [12]. In this study, we set up to determine whether bpV(pic) exerted its neuroprotective effect in cerebral ischemia injury through both PTEN inhibition and ERK 1/2 activation. We indicate that the neuropective effect of bpV(pic) get through by activation of AKT and ERK 1/2, and the bpV(pic) activation of AKT dependent on inhibition of PTEN lipid phosphatase activity only, activation of ERK 1/2 get through by both inhibition of PTEN and independent of PTEN (Fig. 8).

Previous studies have demonstrated that the AKT activity can be increased by bpV(pic) [49]. Our finding confirms that the bpV(pic) enhences the AKT activity through the PTEN lipid activity inhibition. After cerebral ischaemia–reperfusion, the p-AKT and p-ERK 1/2 were down-regulated, inducing the increase of neuronal death and cerebral injury. After treated with bpV(pic), results show a positive improvement in the above phenomenon. But we found that the p-ERK 1/2 activity also increased after injected bpV(pic). As previous reports show that ERK 1/2 activation also have a neuroprotective effect [50, 51]. A number of pathways, growth factors and hormones are shown to confer protection through up-regulation of ERK 1/2 activity [52, 53]. Our data shows that bpV(pic) induced neuroprotection after ischemic stroke also depends on enhancement of ERK 1/2 activity, supports the ERK 1/2 neuroprotective role in ischaemic stroke. BpV(pic) always perform as a inhibitor of PTEN to inhibit PTEN lipid phosphatase activity. Thus, we further inverstigated the mechanism of bpV(pic) increasing ERK 1/2 activity. We found that bpV(pic) enhances ERK 1/2 activity not only by inhibiting of PTEN lipid phosphatase activity, but also by increasing of ERK 1/2 activity independently of PTEN. Our data also shows that the bpV(pic) is not directly affect the activity of ERK 1/2. As reported previously, ERK 1/2 has many up-stream signaling, like MEK, Raf, PKA and etc [54–56]. Thus, it is possible that ERK 1/2 activation by bpV(pic) might depend on PKA or others. The underlying mechanisms remain to be inverstigated. Furthermore, it is known that BpV(pic) inhibits the dephosphorylation of autophosphorylated insulin receptors [57, 58]. As phosphorylation of ERK1/2 is intimately connected to insulin signalling [59, 60]. Therefore, when we use bpV(pic) to act on brain insulin receptors, we should consider the effects of ERK 1/2 signaling.

Our finding demonstrates that bpV(pic) can activate AKT activity through the inhibition of PTEN lipid phosphatase activity. Interestingly, we show that ERK 1/2 can be also activated by bpV(pic). The bpV(pic)-induced ERK 1/2 activation is not only through the inhibition of PTEN lipid phosphatase activity, but is also independent of PTEN inhibition. Consequently, bpV(pic)-induced neuroprotection in ischemic stroke is depend on the inhibition of PTEN lipid phosphatase activity and the enhancement of ERK 1/2 activity that is independent of PTEN inhibition. Therefore, the effect of bpV(pic) on ERK 1/2 signaling should be considered while using bpV(pic) as a PTEN inhibitor in experimental conditions.

## Electronic supplementary material

Below is the link to the electronic supplementary material.

Supplementary material 1 (TIF 1285 KB)

Supplementary material 2 (TIF 653 KB)

Supplementary material 3 (TIF 861 KB)
