# Supplementary figures and images for: ERK 1/2 Activation Mediates the Neuroprotective Effect of BpV(pic) in Focal Cerebral Ischemia–Reperfusion Injury
Source: Neurochem Res. 2018 Jun 7;43(7):1424–38. doi: 10.1007/s11064-018-2558-z (PMC6006215; doi:10.1007/s11064-018-2558-z)

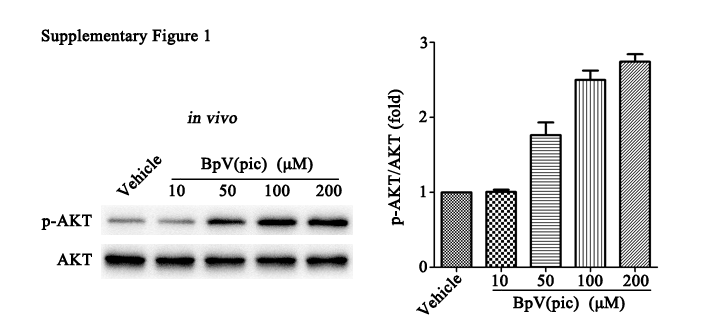

Supplement: Supplementary file 1 — Supplementary material 1 (TIF 1285 KB) [file 11064_2018_2558_MOESM1_ESM.tif]

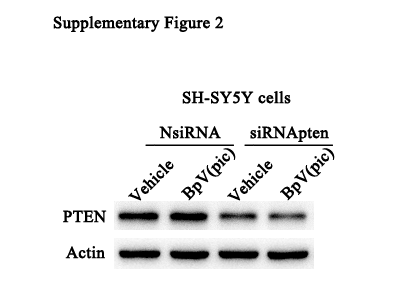

Supplement: Supplementary file 2 — Supplementary material 2 (TIF 653 KB) [file 11064_2018_2558_MOESM2_ESM.tif]

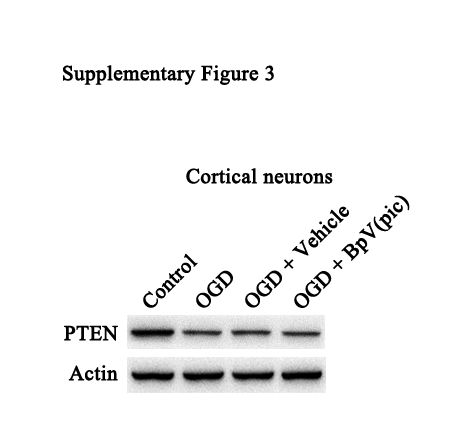

Supplement: Supplementary file 3 — Supplementary material 3 (TIF 861 KB) [file 11064_2018_2558_MOESM3_ESM.tif]
